# Supplementary material for: Using unofficial media, less trusting of Chinese polity?—An analysis based on the moderated mediation effect
Source: PLoS One. 2023 Jun 28;18(6):e0286985. doi: 10.1371/journal.pone.0286985 (PMC10306219; doi:10.1371/journal.pone.0286985)
Supplement: Attachment — Submitted filename: 7 March review China.docx [file pone.0286985.s002.docx]

**Using Unofficial Media, Less Trusting of Polity? Evidence from China**

1. **Due to the increasing influence of unofficial media in China, learning how to build political trust is crucial to advancing the country's system of national governance is the main point of discussion of this paper. But In the abstract section I am not clear with the findings and implications of the study.**
2. **And the study combines the bootstrap method to construct a mixed model with subjective well-being as intermediary variable and official media use as the moderating variable to empirically explore the impact of unofficial media use on political trust and its mechanism.**

**I would like to know the novelty of this research work.**

3. **In the introduction section please write something about why media is seen as playing a central role. Background of the study is missing. Include some discussion about China's media system as well. Discuss the impact of both mainstream and alternative media on popular perceptions of political philosophies.**
4. **The theoretical assumptions regarding how this official media use can be used as a moderating variable to empirically study the impact of unofficial media use on political trust and its mechanism leave me unsatisfied as well.**
5. **What new insights this manuscript adds to the existing literature?**
6. **It would be helpful if you could deepen the development of the theoretical model and provide additional literature for testing hypotheses.**
7. **Clarify the sample design and selection part.**
8. **This is a study in respect to China's background. What if we extrapolate these findings and apply them to other nations?**
